# 

**Published:** 1869-05

**Authors:** 


### Books and Pamphlets Received.

A Treatise on the Function of Digestion; its Disorders and their Treatment. By F. W. Pavy, M. D., F. R. S., Fellow of the Royal College of Physicians; Senior Assistant Physician to and Lecturer on Physiology at Guy's Hospital. From the second London edition. Philadelphia: Henry C. Lea, 1869. Received through and for sale by Breed & Lent, Buffalo.

Two Prize Essays on the Physical Indications of Longevity. Written by J. V. C. Smith, M. D. of Boston, and J. H. Griscom, M. D. of New York, for the American Popular Life Insurance Company. New York: Wm. Wood & Co., 1869.

Proceedings of the State Medical Society of Michigan, for the year 1867 and '68.

